# Bumetanide increases Cl^-^-dependent short-circuit current in late distal colon: Evidence for the presence of active electrogenic Cl^-^ absorption

**DOI:** 10.1371/journal.pone.0171045

**Published:** 2017-02-02

**Authors:** Lieqi Tang, Xiefan Fang, Steven P. Winesett, Catherine Y. Cheng, Henry J. Binder, Scott A. Rivkees, Sam X. Cheng

**Affiliations:** 1 Department of Pediatrics, University of Florida, Gainesville, FL, United States of America; 2 Department of Internal Medicine, Yale School of Medicine, New Haven, CT, United States of America; 3 Department of Pediatrics, Gastroenterology, Hepatology, and Nutrition, University of Florida, Gainesville, FL, United States of America; Universidad de la Laguna, SPAIN

## Abstract

Mammalian colonic epithelia consist of cells that are capable of both absorbing and secreting Cl^-^. The present studies employing Ussing chamber technique identified two opposing short-circuit current (I_sc_) responses to basolateral bumetanide in rat distal colon. Apart from the transepithelial Cl^-^-secretory I_sc_ in early distal colon that was inhibited by bumetanide, bumetanide also stimulated I_sc_ in late distal colon that had not previously been identified. Since bumetanide inhibits basolateral Na^+^-K^+^-2Cl^-^ cotransporter (NKCC) in crypt cells and basolateral K^+^-Cl^-^ cotransporter (KCC) in surface epithelium, we proposed this stimulatory I_sc_ could represent a KCC-mediated Cl^-^ absorptive current. In support of this hypothesis, ion substitution experiments established Cl^-^ dependency of this absorptive I_sc_ and transport inhibitor studies demonstrated the involvement of an apical Cl^-^ conductance. Current distribution and RNA sequencing analyses revealed that this Cl^-^ absorptive I_sc_ is closely associated with epithelial Na^+^ channel (ENaC) but is not dependent on ENaC activity. Thus, inhibition of ENaC by 10 μM amiloride or benzamil neither altered the direction nor its activity. Physiological studies suggested that this Cl^-^ absorptive I_sc_ senses dietary Cl^-^ content; thus when dietary Cl^-^ was low, Cl^-^ absorptive I_sc_ was up-regulated. In contrast, when dietary Cl^-^ was increased, Cl^-^ absorptive I_sc_ was down-regulated. We conclude that an active Cl^-^ extrusion mechanism exists in ENaC-expressing late distal colon and likely operates in parallel with ENaC to facilitate NaCl absorption.

## Introduction

Sodium chloride transport in mammalian colon occurs in two opposing directions: secretion and absorption. The secretory process is primarily localized to crypt cells, whereas the absorptive process is mainly present in surface cells [1, 2]. During secretion, basolateral bumetanide-sensitive Na^+^-K^+^-2Cl^-^-cotransporter (NKCC1) moves Cl^-^ uphill into the cell using the Na^+^ electrochemical gradient generated by Na^+^,K^+^-ATPase (NKA); Cl^-^ is then transported out of the cell into the lumen via apical Cl^-^ channels such as cystic fibrosis transmembrane conductor regulator (CFTR), and Na^+^ flows through a paracellular shunt [2]. In this secretory process, the transport of Cl^-^ anion is active and can be measured in an Ussing chamber as a decrease in bumetanide-sensitive short-circuit current (I_sc_), and the transport of the counter ion Na^+^ is passive, i.e., it only follows the transepithelial voltage, V_T_, set up by CFTR-mediated Cl^-^ secretion, and is therefore not electrogenic and will not be reflected in I_sc_ measurement [2].

On the contrary, Na^+^ and Cl^-^ movements across absorptive surface epithelium are believed to be opposite to secretion, both in direction and in sequence. Na^+^ is taken up from the luminal side of the epithelium by the amiloride-sensitive epithelial Na^+^ channel (ENaC), driven by the Na^+^ electrochemical gradient generated by the NKA, and Na^+^ is then pumped out of the cell by the basolateral NKA, and Cl^-^ flows passively by electrodiffusion [2]. The latter occurs either paracellularly or through a transcellular shunt pathway along a downhill driving force for Cl^-^ [2]. In this absorptive process, the movement of Na^+^ is active and electrogenic, and can be measured in an Ussing chamber as amiloride-sensitive I_sc_ decrease, whereas the movement of Cl^-^ is considered passive and is solely dependent on the lumen-negative V_T_ generated by ENaC.

However, although passive Cl^-^ absorption may occur when luminal Cl^-^ concentration ([Cl^-^]_L_) is high, [Cl^-^]_L_ in the colon is variable, and depends on the colonic secretory rate, the Cl^-^ content in the diet, and the luminal concentrations of other anions. Under normal non-diarrheal conditions, [Cl^-^]_L_ in the colon is low because, under these conditions, the main luminal anions are short-chain fatty acids (SCFAs), which may have a concentration as high as 120 mM [3, 4]. Furthermore, as fluid passes along the length of colon, [Cl^-^]_L_ falls further (due to reabsorption), and when it reaches the distal colon, particularly the late distal colon, the [Cl^-^]_L_ can become extremely low (e.g., ≤ 10 mM [5], which is far below the estimated intracellular [Cl^-^] of 30–40 mM [6–8]). Therefore, under these ionic conditions passive absorption of Cl^-^ will not occur. This raises the question of whether an active mechanism for Cl^-^ absorption exists in the distal colon.

According to the aforementioned current transport model, one would expect that luminal lose dose amiloride application would inhibit ENaC and thereby reduce I_sc_ in late distal colon. This is because ENaC-mediated electrogenic Na^+^ absorption is restricted to the surface epithelium in this portion of the colon [2]. One would also anticipate that serosal bumetanide treatment would inhibit NKCC1 and thereby reduce I_sc_ in both early and late distal colon, as NKCC1 that mediates the electrogenic Cl^-^ secretion is primarily located in crypt cells, which are present in both early and late distal colon [2, 9].

Therefore, in the present study our initial experiments were performed to examine amiloride- and bumetanide-sensitive I_sc_ responses in early and late distal colonic mucosa of rats using Ussing chamber technique [10]. We found that mucosal amiloride inhibited I_sc_ in the late distal colon and that serosal bumetanide inhibited I_sc_ in the early distal colon as predicted. Surprisingly, however, in the late distal colon serosal bumetanide caused no inhibition but I_sc_ stimulation. This positive I_sc_ response to bumetanide in the late distal colon cannot be explained by the aforementioned Cl^-^ secretory I_sc_ as predicted by the current model [2]; instead, it is consistent with the presence of an active Cl^-^ absorptive current in this portion of the colon. In agreement with this, our ion dependence studies demonstrated that this positive or stimulatory I_sc_ by bumetanide in the late distal colon was indeed a Cl^-^ (not Na^+^ or K^+^) current, and prior inhibition of ENaC with amiloride or benzamil, which diminished the lumen-negative V_T_, was unable to diminish this stimulatory I_sc_ by bumetanide. Thus, these findings confirm that this Cl^-^ absorptive I_sc_ is neither passive nor dependent on ENaC-mediated Na^+^ absorption, as previously predicted. In an attempt to define the apical and basolateral transporters involved, in subsequent experiments we further characterized this electrogenic Cl^-^ absorptive I_sc_, both pharmacologically using transport inhibitors and molecularly using gene differential expression analysis combined with Western blots. We also performed physiological experiments that respectively increase and decrease dietary Cl^-^ in order to explore the physiological role this electrogenic Cl^-^ absorptive I_sc_ may play in this salt-sensitive late colonic epithelium and to determine if it is up-regulated in response to decreased dietary Cl^-^ and down-regulated in response to increased dietary Cl^-^. Together, our data established that an active Cl^-^ absorptive mechanism is present in late distal colon in parallel with ENaC, and that it is actively involved in NaCl absorption when salt is in deficit.

## Materials and methods

### Animals and tissue preparation

Experiments were performed with non-fasting male adult Sprague-Dawley rats (5–10 week old with a body weight of 100–300 g) obtained from Charles River Laboratories. Before sacrifice, animals were subjected to one of the following three dietary treatments for a week: 1) normal salt diet (0.3% sodium and 0.5% chloride; Harlan Teklad, Madison, WI, USA; cat. no. T7012) plus tap water in drinking bottle, 2) low salt diet (0.1% sodium and 0.2% chloride; Harlan Teklad, Madison, WI, USA; cat. no. T7034) plus tap water in drinking bottle, 3) normal salt diet plus normal saline (0.9% NaCl) in drinking bottle to enhance daily salt intake. Animals were sacrificed with standard CO_2_ inhalation, followed by cervical dislocation. A segment of distal colon of approximately 4–5 cm in length between the two landmark lymph nodes was excised and divided into early distal colon (the segment in proximity to the proximal lymph node), and late distal colon (the segment adjacent to the distal lymph node). The late distal colon was located approximately 1 cm from the anus. The use of animals, as well as the protocol for isolating colon tissues, was approved by the Institutional Animal Care and Use Committee (IACUC# 201507567) at the University of Florida.

### Ussing chamber and short-circuit current measurements

Isolated colonic segments were rinsed with physiological Ringer solutions, and cut open along the mesenteric border, and then the serosa and muscular layers were stripped as described [11]. The resulting mucosal sheets were mounted on tissue-holding slides (window area = 0.3 cm^2^), placed in an Ussing chamber (Physiologic Instruments, San Diego, CA), and perfused with indicated Ringer solutions, gassed with 5% CO_2_ / 95% O_2_ when HCO_3_^-^-containing solution was used or 100% O_2_ when HCO_3_^-^-free solution was used. Each side of the chamber contained 3 ml of solution and the temperature of the solution was adjusted to and maintained at 37°C by heated water-jacketed reservoirs. To facilitate comparison, in some experiments the mucosa segment was also divided longitudinally into 2 equal pieces and mounted into two separate Ussing chambers, with one piece of mucosa being used as control and the other as treatment. Tissues were voltage-clamped to zero potential difference by the application of short-circuit current (I_sc_) (Voltage-Current Clamp, VCC MC8; Physiologic Instruments, San Diego, CA), except for brief interruption at 20-second intervals for recording of open-circuit potential (*V*_*T*_, mV). Following the establishment of baseline and basal recordings, tissues were treated with bumetanide (100 μM, basolateral or apical), amiloride (10 μM, apical), barium (5 mM, apical), forskolin (500 nM, basolateral) or other indicated inhibitors, and I_sc_ responses were recorded. I_sc_ was measured in microamperes (μA) and is expressed as μA/cm^2^. Tissue resistance (R, Ω.cm^2^) was calculated from Ohm’s law.

Under the present experimental conditions, the changes in I_sc_ (ΔI_sc_) in these distal colonic segments are largely reflective of electrogenic Cl^-^, Na^+^ and K^+^ movements. In this study, ΔI_sc_ refers to induced steady state responses before and after drug treatment. Unless otherwise stated, ΔI_sc_^Bumetanide^ was defined as ΔI_sc_ responses to 100 μM serosal bumetanide. ΔI_sc_^Amiloride^ was defined as ΔI_sc_ responses to 10 μM mucosal amiloride. ΔI_sc_^Barium^ was defined as ΔI_sc_ responses to 5 mM mucosal barium. ΔI_sc(HCO3)_^Forskolin^ was defined as ΔI_sc_ responses to 500 nM serosal forskolin, measured in the presence of 100 μM serosal bumetanide (to inhibit Cl^-^ current), 10 μM mucosal amiloride and 5 mM mucosal barium (to selectively inhibit Na^+^ and K^+^ conductance). These transport inhibitors were employed to reduce current interference from non HCO_3_^-^ conductance as described elsewhere [11–13] and to help determine the magnitude of apical CFTR anion conductance as, under present experimental conditions, forskolin-induced I_sc_ primarily reflects HCO_3_^-^ secretion mediated by apical CFTR and basolateral Na^+^-HCO_3_^-^ cotransporter [11, 14]. A pilot study using HCO_3_^-^-free Ringer solution confirmed its primary HCO_3_^-^ dependency [mean ± SE (n) μA/cm^2^ early and late distal colon ΔI_sc(HCO3)_^Forskolin^ in presence *vs*. absence of HCO_3_^-^ ion: 29.3 ± 4.0 (8) and 12.3 ± 1.6 (8) *vs*. 4.6 ± 0.4 (3) and 2.6 ± 2.4 (3)**, P<0.01].

To determine ion dependency, additional experiments were performed in Cl^-^-free, HCO_3_^-^-free, or Na^+^-free Ringer solutions. In those experiments, tissues were bathed initially in normal Ringer solutions and then in respective Cl^-^/HCO_3_^-^/Na^+^-free Ringer solutions, as described [15]. I_sc_ were recorded and corrected for junction potentials before used for calculations.

### Illumina transcriptomic RNA sequencing (RNA-Seq) and differential gene expression analysis

RNA-Seq libraries were prepared as described [16]. mRNA was isolated from total RNA prepared from early and late colonic mucosa of rats using NEXTflex™ Poly(A) Beads (Bioo Scientific, Austin, TX, USA). Sequencing libraries were prepared with the NEBNext® mRNA Library Prep Master Mix Set for Illumina (NEB, Ipswich, MA, USA) and the NEBNext Multiplex Oligos for Illumina (NEB). Illumina-adapted libraries were pooled at equal molar ratio and sequenced with one High Output 1×75 cycles run on a NextSeq500 sequencer (Illumina, San Diego, CA, USA).

The fastq files generated from RNA-Seq were uploaded to the UF Research Computing Galaxy instance developed by the University of Florida [16]. The data were cleaned with the FastQC program and mapped to the rat genome (rn6) with the Tophat2 tool. Differential expression (DE) of genes between early and late colonic mucosa segments was analyzed using Cufflinks [17], with Ensembl Rattus_norvegicus.Rnor_6.0.84.gtf as the reference annotation. Genes with false discovery rate (FDR) less than 0.05 and absolute fold change greater than 1.5 were considered as significant.

### Western blots

Isolated early and late distal rat colonic mucosa were rinsed in Ringer buffer and lysed as previously described [18]. The lysates were briefly sonicated and were incubated at room temperature for 15 min before they were loaded onto a 6% SDS-polyacrylamide gel. After electrophoresis, proteins were transferred to nitrocellulose membranes (Bio-Rad) by electro blotting. After protein transfer, wet membranes were stained with ponceau S (Amresco, OH) for 1 minute and quickly destained in water to remove non-specific ponceau staining. The membranes were then imaged and total protein quantified using Imagelab 4.1 (Bio-Rad). After destaining in Tris-buffered saline with 0.1% Tween 20 (TBST), membranes were quenched with 5% nonfat milk in TBST for 1 h at room temperature. Expression of KCC1 protein was detected with an affinity-purified polyclonal antibody raised against a 13-amino acid peptide corresponding to amino acid residues 998–1010 of the COOH-terminus of rat KCC1 (Alomone Labs, Israel, Cat #AKT-001). Incubation with primary antibody was made overnight in 5% nonfat milk containing TBST (1:200 dilution). After three 20-min washes at room temperature in TBST, membranes were incubated with anti-rabbit IgG secondary antibody conjugated horse radish peroxidase (1:5,000, Sigma, MO) for 1 h. Following three washes at room temperature in TBST, membranes were incubated with Clarity chemiluminescence substrate (Bio-Rad), imaged on the Chemidoc MP, and the bands detected analyzed with Imagelab 4.1. The KCC1 protein signals were normalized by ponceau S staining as a loading control as described [19]. Differential expression of KCC1 between early and late colonic mucosa segments was analyzed and expressed as fold change (late distal colon/early distal colon).

### Chemicals, reagents and solutions

Polyclonal anti-KCC1 antibody was purchased from Alomone Labs, Israel. Bumetanide, amiloride, benzamil, barium, glibenclamide, 5-nitro-2-(3-phenylpropylamino)benzoic acid (NPPB), CFTR(inh)-172, and forskolin were obtained from Sigma while GlyH 101 was from Calbiochem. All stock solutions were prepared in DMSO. Normal salt diet and low salt diet were purchased from Harlan Teklad. Ringer solution contains (in mM) 140.8 Na^+^, 5.2 K^+^, 123.5 Cl^-^, 25 HCO_3_^-^, 1.25 Ca^2+^, 1.25 Mg^2+^ and 5 glucose, pH 7.4. Cl^-^-free Ringer solution contains HCO_3_^-^ (25 mM) with no Cl^-^. HCO_3_^-^-free Ringer solution contains Cl^-^ with no HCO_3_^-^. Na^+^-free Ringer solution contains no Na^+^. In these solutions, isethionate was used in place of anion Cl^-^ and HCO_3_^-^ and N-Methyl-D-glucamine (NMDG) was used as a substitute for Na^+^. HCO_3_^-^-containing solutions were gassed with 95% O_2_-5% CO_2_; HCO_3_^-^-free solution was gassed with 100% O_2_.

### Statistical analysis

Values are expressed as means ± S.E.M. ΔI_sc_ refers to induced steady state responses before and after drug treatment. Data were analyzed by the paired or unpaired two-tailed Student's *t*-test, as appropriate, using Microsoft Excel 2010 for Windows or GraphPad Prism version 6 for Windows (GraphPad Software, San Diego, CA). *P* < 0.05 was considered significant.

## Results

### Bumetanide induces two opposing I_sc_ in distal colon: Inhibitory in early distal colon and stimulatory in late distal colon

The initial set of experiments was performed to assess transepithelial Cl^-^ secretion in the distal colon of rats by measuring short-circuit current (I_sc_) response to basolateral bumetanide of colonic mucosa mounted in Ussing chambers. As a control, the effect of apical bumetanide was examined.

**Fig 1:**
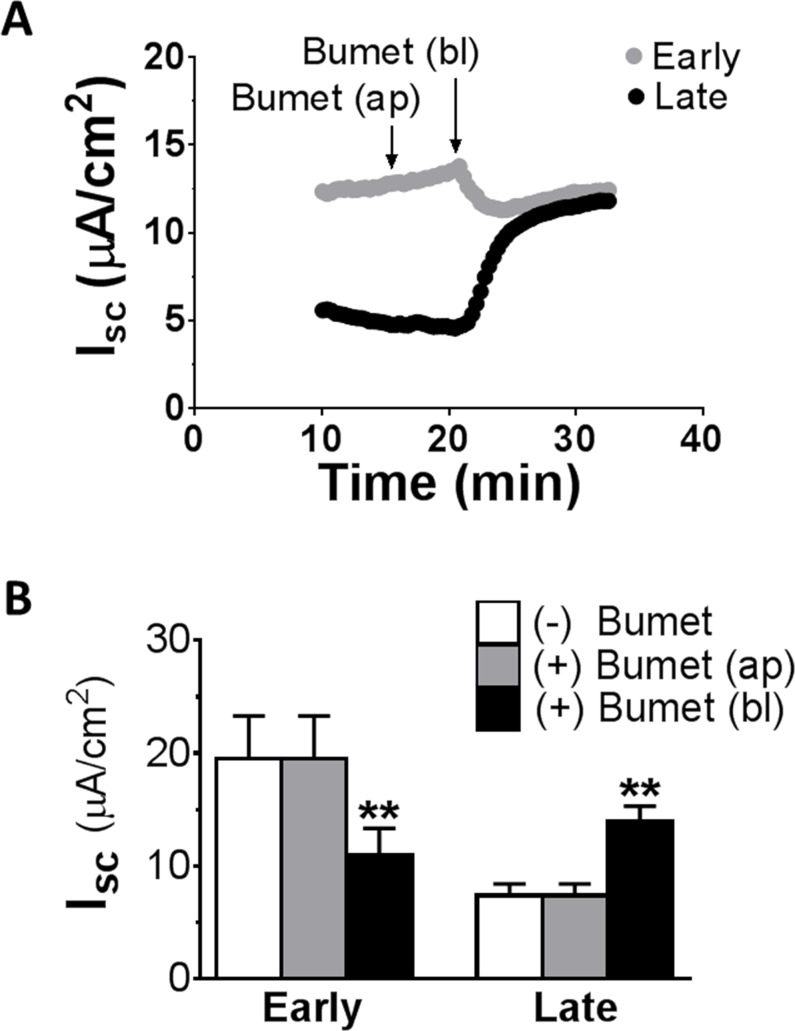
I_sc_ responses to apical and basolateral bumetanide in early and late distal colon of rats. Measurements were made in normal Ringer solution. Note that while apical bumetanide (100 μM) had no effect on either colonic segment, basolateral bumetanide (100 μM) induced two opposite I_sc_ responses: inhibitory in early but stimulatory in late distal colon. ap, apical; bl, basolateral. ** P<0.01 *vs*. absence of bumetanide using paired *t* test. N = 10.

Fig 1 summarizes the effects of bumetanide on I_sc_ in early and late distal colon. Fig 1A shows representative I_sc_ responses to sequential additions of bumetanide (100 μM) to apical and basolateral chambers in early and late distal colon. Quantitative summaries are shown in Fig 1B. Without bumetanide, I_sc_ was significant larger in early than late distal colon (Table 1). The addition of bumetanide to the apical side of the epithelium had no effect on I_sc_ in either colonic segment. In contrast, the addition of bumetanide to the basolateral side significantly decreased I_sc_ in early distal colon, as predicted by the transport model for active secretion of Cl^-^ [2]. Surprisingly, in late distal colon, the same basolateral bumetanide treatment caused a significant I_sc_ increase, rather than the anticipated I_sc_ decrease. Thus, it appears that an unusual electrogenic ion transport mechanism is present in late distal colon.

**Table 1:** Basal bioelectric parameters in rat early and late distal colon.

	Early distal colon	Late distal colon
I_sc_, μA/cm^2^	65.0 ± 12.7 (10)	24.7 ± 3.3 (10)**
R, Ω.cm^2^	70.9 ± 11.6 (10)	122.7 ± 15.3 (10)**
PD, mV	5.8 ± 1.1 (10)	4.5 ± 0.9 (10)

Data shown are means ± SEM (n).

** P<0.01 *vs*. Early distal colon.

### The stimulatory I_sc_^Bumetanide^ colocalizes with ENaC-mediated electrogenic Na^+^ absorptive I_sc_ and K^+^ secretory I_sc_ but does not depend on their activities

**Fig 2:**
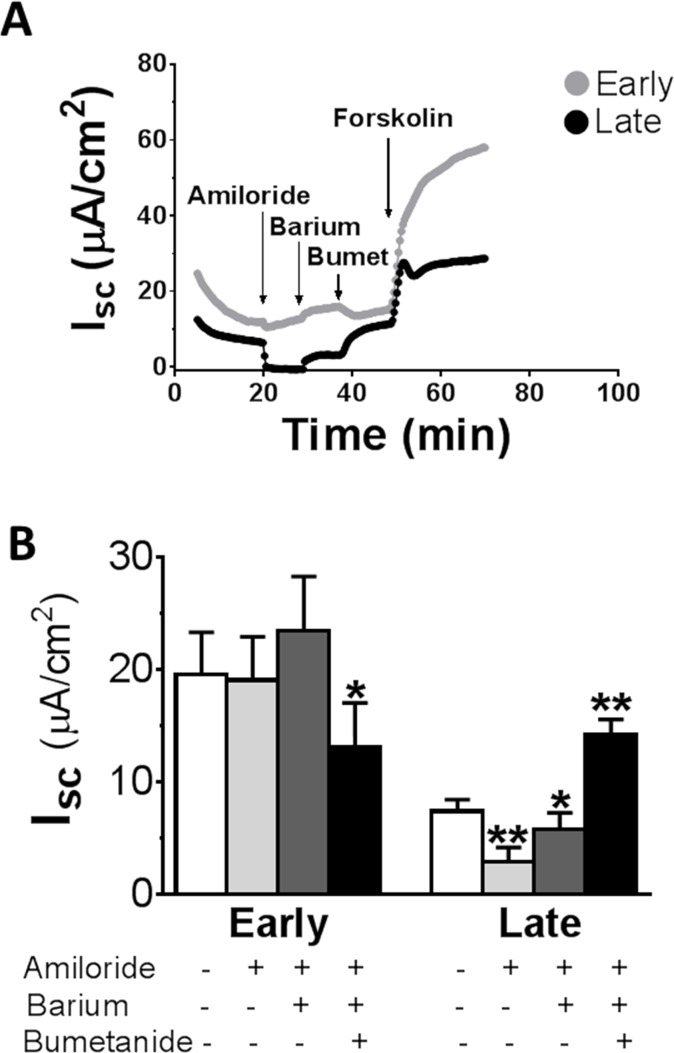
I_sc_ responses to basolateral bumetanide in the presence of amiloride and barium. I_sc_ was measured in normal Ringer solution. Note that amiloride (10 μM, apical) significantly decreased and barium (5 mM, apical) significantly increased I_sc_ in late distal colon. However, these treatments did not alter the subsequent I_sc_ responses to bumetanide (100 μM, basolateral) in either colonic segment. Subsequent addition of forskolin (500 nM, basolateral) caused I_sc_ stimulation in both segments (see text). * P<0.05 and ** P<0.01 *vs*. prior treatment by paired *t* test. N = 10.

Apart from actively secreting Cl^-^, the distal colon, particularly the late distal colon, also actively absorbs Na^+^ and secretes K^+^. It is, therefore, possible that the stimulatory I_sc_^Bumetanide^ noted in late distal colon might be a Na^+^ absorptive current, a K^+^ secretory current, or both. NKCC1 mediates the influx of Na^+^, K^+^, and 2Cl^-^, utilizing the inward gradient for Na^+^ for the uphill transport of two Cl^-^. Thus, inhibition of NKCC1 by bumetanide not only decreases Cl^-^ secretion, but may also reduce cellular Na^+^ and K^+^; the resultant reduction of cellular Na^+^ and K^+^ may result in stimulation of ENaC and blockage of spontaneous K^+^ secretion, leading to an increase in I_sc_. To explore these possibilities, we examined I_sc_ responses to basolateral bumetanide following inhibition of the activities of apical ENaC and K^+^ secretion by amiloride and barium, respectively (Fig 2). Indeed, under present experimental conditions electrogenic Na^+^ absorption and K^+^ secretion were present in the late distal colon, as evidenced by amiloride-induced decrease of I_sc_ and barium-induced increase of I_sc_ (Fig 2A). However, prior inhibition of these ion transporters did not significantly alter the bumetanide-induced I_sc_ responses, neither in early nor late distal colon (Fig 2A; quantitative summary in Fig 2B), thus excluding the contribution of Na^+^ absorptive or K^+^ secretory current.

Since the lack of effect of amiloride was not anticipated, a 10x higher dose (100 μM) amiloride was used to be certain that inhibition of ENaC activity was complete. We also tested the effect of benzamil, a more potent ENaC inhibitor. The I_sc_ responses to bumetanide remained unaltered with either inhibitor (Table 2). Since the dose of 100 μM amiloride used also inhibits Na^+^/H^+^ exchange, the lack of effect of the latter suggests that Na^+^/H^+^ exchange is not also contributing.

**Table 2:** Effects of amiloride and benzamil on ΔI_sc_^Amiloride^ and ΔI_sc_^Bumetanide^ in rat early and late distal colon*.

	ΔI_sc_^Amiloride^, μA/cm^2^		ΔI_sc_^Bumetanide^, μA/cm^2^	
	Early distal colon	Late distal colon	Early distal colon	Late distal colon
None			-17.4 ± 3.1 (10)	22.3 ± 3.8 (10)
Amiloride (10μM)	-1.5 ± 0.7 (10)	-6.2 ± 6.8 (10)	-26.2 ± 5.1 (10)ns	27.2 ± 6.4 (10)ns
Amiloride (100μM)	-2.2 ± 0.8 (4)NS	-10.0 ± 7.3 (4)NS	-17.0 ± 2.0 (4)ns	37.0 ± 11.0 (4)ns
Benzamil (5μM)	-1.8 ± 0.8 (4)NS	-6.3 ± 3.8 (4)NS	-12.0 ± 4.0 (4)ns	33.0 ± 2.0 (4)ns

* ΔI_sc_^Amiloride^ refers to induced ΔI_sc_ before and after indicated drug treatment. Data shown are means ± SEM (n). ns P>0.05 *vs*. None. NS P>0.05 *vs*. Amiloride (10μM).

### Bumetanide stimulation of I_sc_ requires Cl^-^ and is inhibited by Cl^-^ channel inhibitors

**Fig 3:**
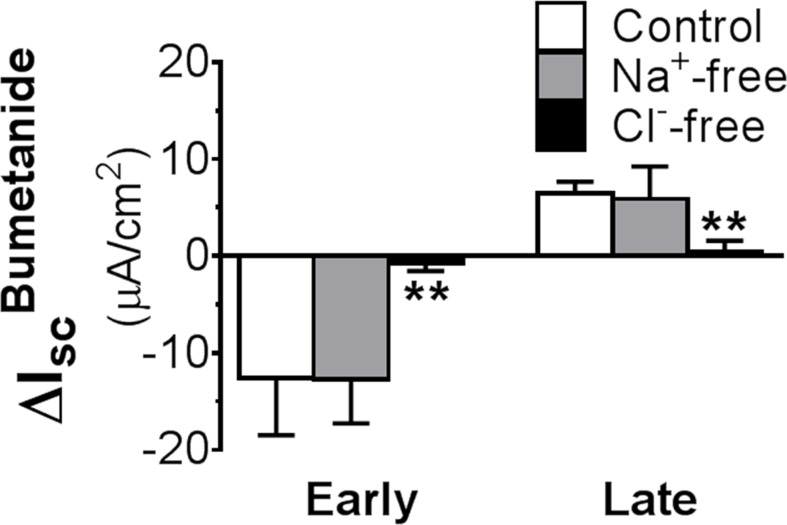
Effects of ion substitution on changes in I_sc_ responses to basolateral bumetanide (100 μM) in early *vs*. late distal colon. Note the two opposing ΔI_sc_^Bumetanide^: negative or inhibitory in early distal colon and positive or stimulatory in late distal colon_._ While Na^+^ substitution caused no significant change in the I_sc_ response in either colonic segment, removal of Cl^-^ from media abolished the I_sc_ responses in both segments. ** P<0.01 *vs*. control. N = 8–12.

The question then arises of whether the positive I_sc_ induced by bumetanide is a Cl^-^ current. To address this, Cl^-^ ion substitution experiments were performed (Fig 3). In these experiments, Cl^-^ was replaced by isethionate. As a control, the effects without Na^+^ were also examined. In the latter case, Na^+^ was replaced by NMDG^+^. In normal Ringer solution, the ΔI_sc_^Bumetanide^ was negative or inhibitory in early distal colon and positive or stimulatory in late distal colon. Neither inhibitory nor stimulatory responses by bumetanide were altered when Na^+^ was removed from Ringer solution. In contrast, both responses to bumetanide were completely abolished when Cl^-^ was eliminated from solution, indicating that, like the inhibitory I_sc_^Bumetanide^, the stimulatory I_sc_^Bumetanide^ is also a Cl^-^ current.

Considering the current direction opposite to the known Cl^-^ secretory I_sc_ and the current location in the salt absorptive late distal colon, it is possible that this positive I_sc_^Bumetanide^ is a Cl^-^ absorptive current. Cl^-^ could be entering the cell down its concentration gradient via apical Cl^-^ channels and subsequently be extruded from the basolateral membrane via the bumetanide-sensitive KCl cotransporter (KCC) using the downhill K^+^ gradient provided by the basolateral Na^+^,K^+^-ATPase (see Discussion below). To address this possibility, we examined the effects of Cl^-^ channel inhibitors NPPB [20] and glibenclamide [21, 22].

**Fig 4:**
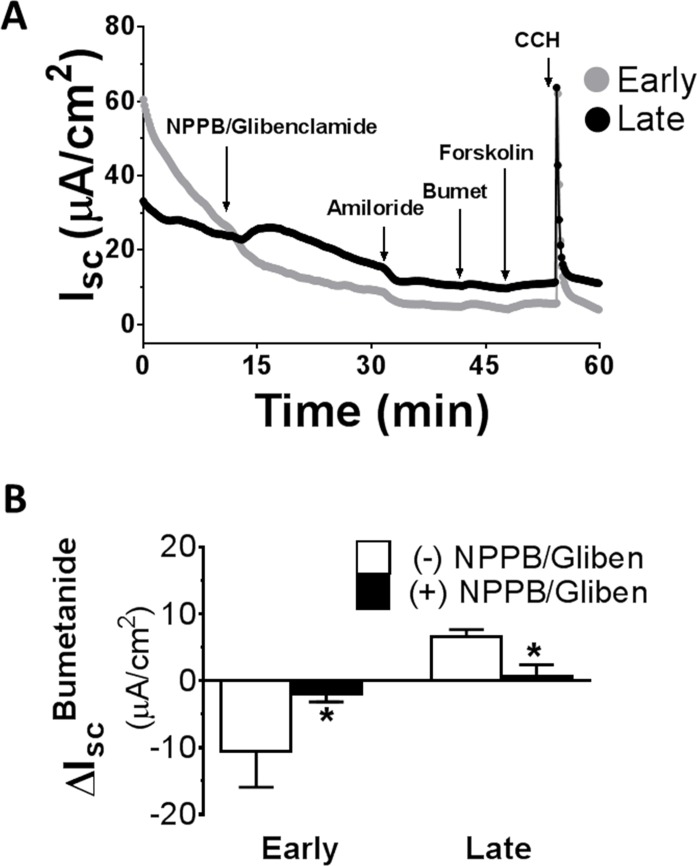
Effects of Cl^-^ channel inhibitors on I_sc_ responses to basolateral bumetanide in early and late distal colon of rats. Measurements were made in normal Ringer solution. Note that pretreatment with NPPB (100 μM, apical) and glibenclamide (300 μM, apical) significantly attenuated I_sc_ responses to bumetanide (100 μM, basolateral) and forskolin (500 nM, basolateral), but not to amiloride (10 μM, apical) or carbachol (CCH, 100 μM, basolateral). **P<0.05 *vs*. (-) NPPB/Glibenclamide. N = 5.

Fig 4 shows effects of NPPB/glibenclamide on I_sc_ responses to bumetanide. As controls, the effects of NPPB/glibenclamide on subsequent I_sc_ responses to forskolin (a measure of cAMP-activated anion channels) and carbachol (a measure of Ca^2+^-activated anion channels) were also assessed. Consistent with the presence of an active Cl^-^ absorptive conductance in the apical membrane of late distal colonic epithelium, the addition of NPPB/glibenclamide to the apical bath increased I_sc_ in late distal colon (Fig 4A). In contrast, the same pretreatment caused an I_sc_ decrease in early distal colon (Fig 4A), in keeping with inhibition of Cl^-^ secretory conductance in the apical membrane of early distal colonic epithelium. Importantly, following NPPB/glibenclamide pretreatment, while the I_sc_ responses to amiloride remained unchanged, the I_sc_ responses to bumetanide was significantly attenuated or abolished (Fig 4A & 4B). NPPB/glibenclamide pretreatment also significantly attenuated the I_sc_ responses to forskolin [compare Fig 4A (presence of inhibitors) with Fig 2A (absence of inhibitors)] but not to carbachol (see Fig 4A). Similar effects were observed with GlyH 101 but not CFTR(inh)-172. This suggests the possible involvement of Cl^-^ conductance in Cl^-^ absorption by late distal colon.

### Effect of bumetanide on transepithelial conductance in distal colon

**Fig 5:**
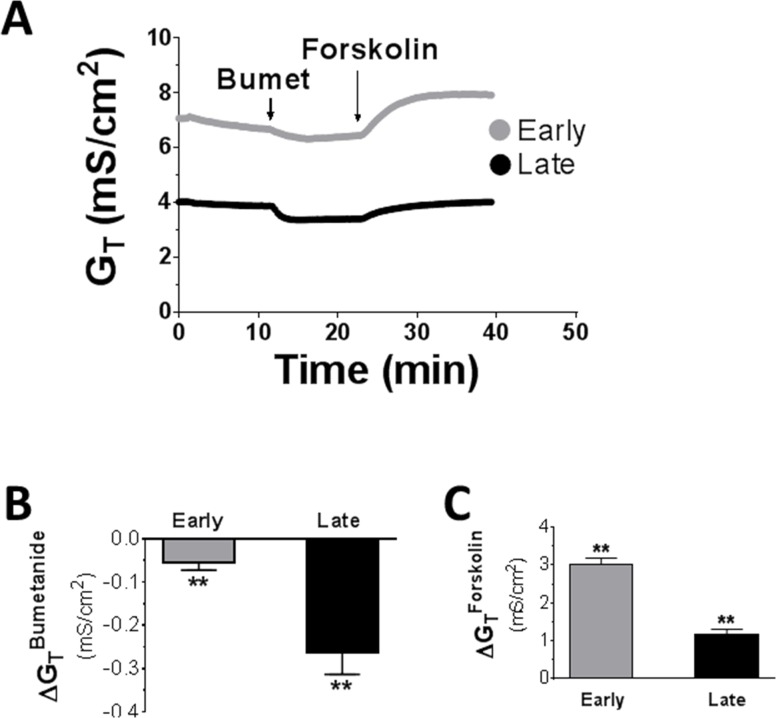
Transepithelial conductance (G_T_) responses to bumetanide (100 μM, basolateral) and forskolin (500 nM, basolateral) in early and late distal colon of rats. The experiments were performed in normal Ringer solution. ** P<0.01 *vs*. prior drug treatment. N = 10.

To provide further evidence that a channel conductance is implicated in Cl^-^ absorptive I_sc_ in late distal colon, effects on transepithelial conductance (G_T_) were examined (Fig 5). If a channel conductance is involved in this transport process, bumetanide treatment should inhibit this channel activity and decrease G_T_. As illustrated in Fig 5A, bumetanide decreased G_T_, not only in early but also in late distal colon. The G_T_ decrease by bumetanide was significantly more pronounced in late than early distal colon (Fig 5B).

**Fig 6:**
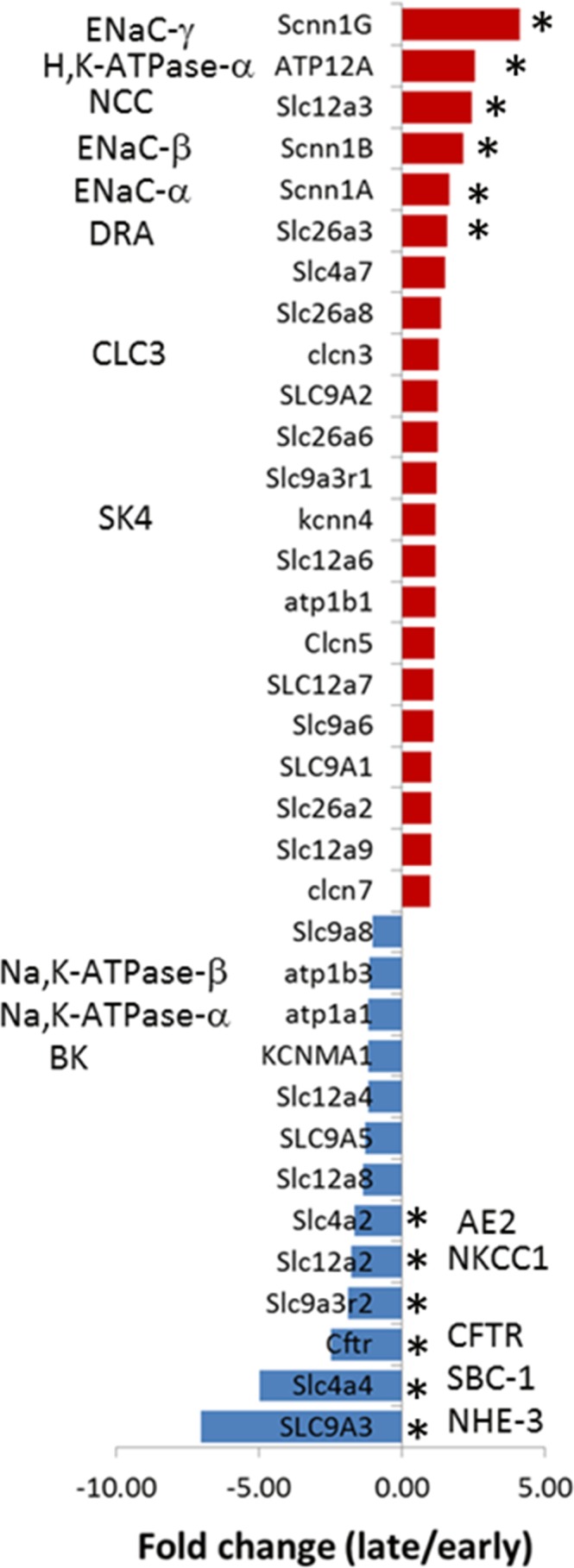
Differential expression of gene transcripts in late distal colon of rats relative to early distal colon. Positive values indicate more abundant transcripts of indexed gene in late than in early distal colon whereas negative values indicate less abundant transcripts of indexed gene in late than in early distal colon. * P<0.05 *vs*. early distal colon.

As a control, the effect of forskolin on G_T_ was also examined (Fig 5A & 5C). Forskolin increased G_T_. In contrast to the pattern induced by bumetanide, the G_T_ increase by forskolin was more pronounced in early than late distal colon_._ The forskolin-induced increases in G_T_ along early and late distal colon are very similar to those found for forskolin-induced I_sc_ (see Fig 2A) and CFTR mRNA (see Fig 6 below), consistent with the known forskolin stimulating effect on CFTR conductance [2].

Together, the results indicate that a transepithelial conductance is inhibited in late distal colon by bumetanide concurrently with transepithelial I_sc_ activation. Based on this and other findings, we concluded that an electrogenic Cl^-^ absorptive mechanism is present in late distal colon. This mechanism involves a NPPB/glibenclamide-sensitive Cl^-^ conductance (likely a CFTR-like Cl^-^ channel/transporter) in the apical membrane, which mediates Cl^-^ entry, and a bumetanide-sensitive Cl^-^ absorbing mechanism (likely a KCl cotransporter) in the basolateral membrane, which mediates Cl^-^ exit.

### Expression of ion transporter in the distal colon

To further characterize this electrogenic Cl^-^ absorptive I_sc_, RNA sequencing analysis was performed on gene transcripts of major ion transporters in rat distal colon (Table 3 & Fig 6). Consistent with the notion that late distal colon differs from early distal colon in salt transport, it was found that transporters associated with salt absorption were expressed in a pattern opposite to transporters responsible for salt secretion; that is, a greater level of expression was noted for ENaC, NCC, H^+^,K^+^-ATPase and CLC in late distal colon when compared with early distal colon segment. On the contrary, a greater level of expression for NKCC1, SBC1, AE2 and CFTR was noted in early distal colon when compared with late distal colon. Interestingly, a similar reciprocal expression pattern was also noted for DRA *vs*. NHE3 in late *vs*. early distal colonic segments. This segregated pattern contrasts with the coupled DRA/NHE3 expression proposed for electroneutral NaCl absorption in the colon [2]. Unlike the transcripts for transporters specific for salt absorption and secretion, the transcripts for Na^+^,K^+^-ATPase (NKA), which is required for both salt absorption and secretion, remained stable in the distal colon. In disagreement with a previous report [23], NKCC2 transcripts were not detected in either colonic segment.

**Table 3:** Differential expression of gene transcripts in rat distal colon.

Transporter	Gene	Late	Early	Fold change (late/early)
ENaC-alpha	*Scnn1A*	251.96	152.597	1.651146*
ENaC-beta	*Scnn1B*	15.9184	7.42909	2.142712*
ENaC-gamma	*Scnn1G*	1.77275	0.43038	4.119034*
Na,K-ATPase-alpha1	*atp1a1*	849.136	974.578	-1.14773
Na,K-ATPase-alpha2	*atp1a2*	0.116211	0.545423	-4.69339*
Na,K-ATPase-alpha3	*atp1a3*	0.031001	0.112315	-3.62297*
Na,K-ATPase-beta1	*atp1b1*	2230.14	1901.33	1.172937
Na,K-ATPase-beta2	*atp1b2*	0.278041	0.621188	-2.23416*
Na,K-ATPase-beta3	*atp1b3*	13.679	15.5672	-1.13804
Na,K-ATPase-gamma	*fxyd2*	2.89002	25.9343	-8.97374*
Na/H exchanger-1	*SLC9A1*	63.3886	60.385	1.049741
Na/H exchanger-2	*SLC9A2*	174.178	136.345	1.27748
Na/H exchanger-3	*SLC9A3*	46.8226	328.108	-7.00747*
NHERF-1	*Slc9a3r1*	420.365	342.149	1.228602
NHERF-2	*Slc9a3r2*	5.00395	9.30693	-1.85992*
Na/H exchanger-5	*SLC9A5*	0.896523	1.13444	-1.26538
Na/H exchanger-6	*Slc9a6*	8.55022	7.7633	1.101364
Na/H exchanger-8	*Slc9a8*	18.4983	19.1032	-1.0327
NKCC2	*Slc12a1*	0	0	
NKCC1	*Slc12a2*	62.0267	109.889	-1.77164*
NCC	*Slc12a3*	8.17396	3.357	2.4349*
KCC1	*Slc12a4*	2.27527	2.63623	-1.15864
KCC2	*Slc12a5*	0.032339	0.029925	1.080664
KCC3	*Slc12a6*	16.8354	14.2482	1.181581
	*SLC12a7*	47.3722	42.1302	1.124424
CCC9	*Slc12a8*	32.1545	43.3327	-1.34764
CCC6/CIP1	*Slc12a9*	27.1636	26.4798	1.025823
	*Slc26a1*	0.348735	0.475976	-1.36486
	*Slc26a2*	96.8234	93.6515	1.033869
DRA	*Slc26a3*	1079.2	673.093	1.603345*
Pendrin	*Slc26a4*	0	0	
Prestin	*Slc26a5*	0	0	
PAT1	*Slc26a6*	6.01403	4.71441	1.27567

* P<0.05 *vs*. early distal colon. Positive values indicate more abundant transcripts of indexed gene in late than in early distal colon whereas negative values indicate less abundant transcripts of indexed gene in late than in early distal colon.

**Fig 7:**
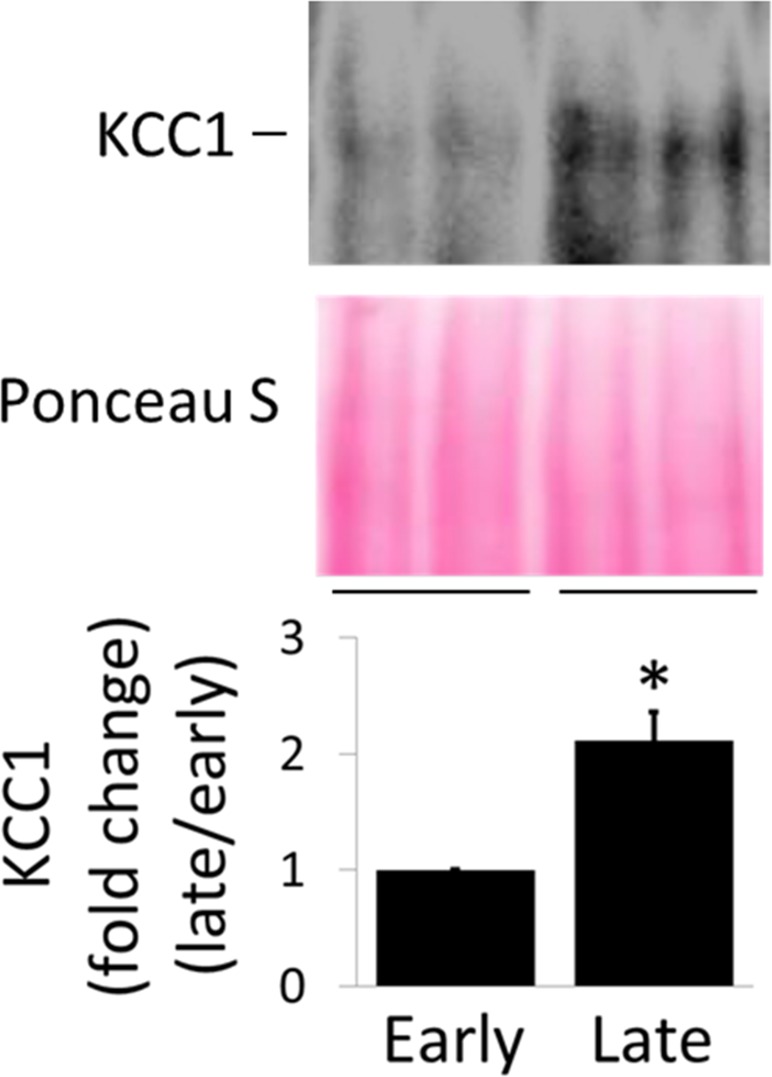
Differential expression of KCC1 protein in late distal colon of rats relative to early distal colon. **Upper panel**, Western blots of KCC1; **Middle panel**, Ponceau S staining; **Lower panel**, The Western blots were quantified and normalized to ponceau S staining. The y-axis represents relative values (changes in fold) with respect to the values of early distal colon. * P<0.05 *vs*. early distal colon. N = 5.

Of note, while transcripts of NKCC1 were found to be differentially expressed within the distal colon and may explain the difference in Cl^-^ secretory I_sc_, such differential distribution was not detected for KCC1 mRNAs. To assess if differential distribution for KCC1 exists at the protein level, Western blot analyses using a specific anti-KCC1 antibody were performed (Fig 7). Approximately two-fold higher KCC1 protein was detected in late than early distal colon.

Together, the results indicate that the segmental heterogeneity in I_sc_ response to bumetanide may represent different patterns of distribution of the two bumetanide-sensitive cation chloride cotransporters, NKCC1 and KCC1. Although NKCC1 and KCC1 both mediate secondary active transport of Cl^-^ through the tight coupling to movement of a cation, they are driven by different electrochemical gradients generated by NKA and therefore result in different outcomes in Cl^-^ homeostasis. Whereas NKCC1 mediates Na^+^ gradient-dependent inward transport of Cl^-^, which increases [Cl^-^]_i_ leading to Cl^-^ secretion, KCC1 mediates K^+^ gradient-dependent outward movement of Cl^-^, which decreases [Cl^-^]_i_ resulting in Cl^-^ absorption. Accordingly, the direction of Cl^-^ transport is determined by the relative expression of these two transporters in segments of distal colon. Since the early distal colon expresses more NKCC1 than KCC1, Cl^-^ secretion observed, whereas the late distal colon expresses more KCC1 than NKCC1 and thus is Cl^-^ absorptive.

### Effects of salt depletion and repletion

**Fig 8:**
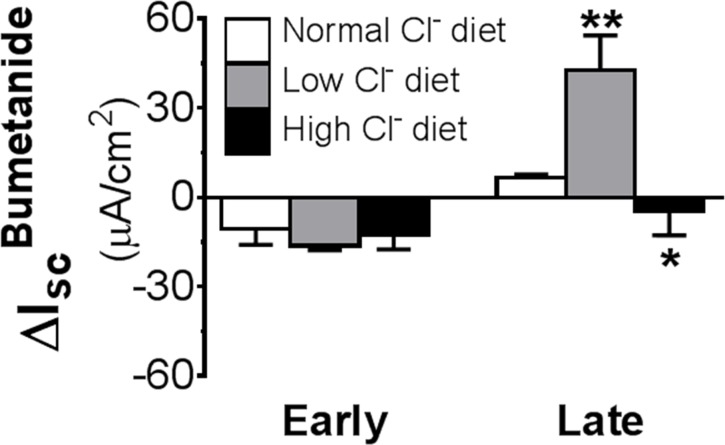
Effects of dietary Cl^-^ depletion and repletion on I_sc_ responses to basolateral bumetanide in early and late distal colon. Note that dietary Cl^-^ depletion enhanced whereas dietary Cl^-^ repletion reduced the positive I_sc_ induced by basolateral bumetanide (100 μM) in late distal colon but had no effect on the induced negative I_sc_ in early distal colon. * P<0.05 and ** P<0.01 *vs*. normal Cl^-^ diet. N = 6–10.

To understand the physiological role this active Cl^-^ absorptive mechanism may play in the salt-sensitive distal colon, experiments that altered NaCl in the diet were performed. In these experiments, animals were subjected to either low or high salt treatment (via diet or drink) for 1 week before colonic I_sc_ responses to bumetanide were determined (Fig 8). As positive control, colonic I_sc_ responses to low dose amiloride were also assessed (Table 4). While no significant changes were found in early distal colon (Fig 8), in late distal colon, decreasing NaCl intake significantly increased the Cl^-^ absorptive I_sc_, whereas increasing NaCl intake significantly decreased it (Fig 8). Similar increases and decreases to low and high salt diets, respectively, were also noted for amiloride-sensitive ENaC current (Table 4). These data established that an active Cl^-^ absorptive mechanism is present in late distal colon in parallel with ENaC, and that it is actively involved in NaCl absorption when salt is in deficit.

**Table 4:** I_sc_^Amiloride^ responses to low and high salt diet treatments in rat distal colon.

	ΔI_sc_^Amiloride^, μA/cm^2^	
	Early distal colon	Late distal colon
Control diet	1.24 ± 0.26 (8)	5.50 ± 1.17 (8)**
Low salt diet	2.92 ± 1.29 (6)#	28.08 ± 6.61 (6)**##
High salt diet	0.12 ± 0.02 (3)##	0.77 ± 0.39 (3)*#

Data shown are means ± SEM (n).

* P<0.05 and ** P<0.01 *vs*. Early distal colon.

## P<0.01and #P<0.05 *vs*. control.

## Discussion

Unlike other extracellular milieu, the lumen of the colon is home to hundreds of trillions of bacteria. There, as high as 120 mM of short-chain fatty acids (SCFA) are normally present due to bacterial fermentation of undigested carbohydrates [3, 4]. Accordingly, in the lumen of the colon, the major anions are SCFA; other anions, such as Cl^-^, are low [5]. For passive Cl^-^ absorption to occur at low [Cl^-^]_L_, apical membrane potential (Ψ_a_) must depolarize markedly to maintain the driving force for Cl^-^ entry into the cell across the apical membrane. Alternatively, intracellular Cl^-^ concentration ([Cl^-^]_i_) must fall appreciably. In the distal colon, while Ψ_a_ depolarizes due to ENaC-mediated electrogenic Na^+^ absorption, it also hyperpolarizes due to spontaneous electrogenic K^+^ secretion. Furthermore, unlike exclusive absorptive epithelia (e.g., sweat duct), colonic epithelia have a more complex task of providing both absorption and secretion of Cl^-^, and [Cl^-^]_i_ may or may not fall in this tissue due to the presence of Cl^-^ entry into the cell from the basolateral NKCC1 [2] or Cl^-^/ HCO_3_^-^ exchanger [13]. Thus, it is not entirely clear whether the changes in Ψ_a_ and [Cl^-^]_i_ are sufficient to maintain the driving force for Cl^-^ influx from the lumen, requisite for passive Cl^-^ absorption to occur in a low [Cl^-^]_L_ situation. It is likely that an alternative active mechanism for Cl^-^ absorption is present in the distal colon, particularly in the late distal colon where, due to reabsorption, [Cl^-^]_L_ is the lowest [5].

**Fig 9:**
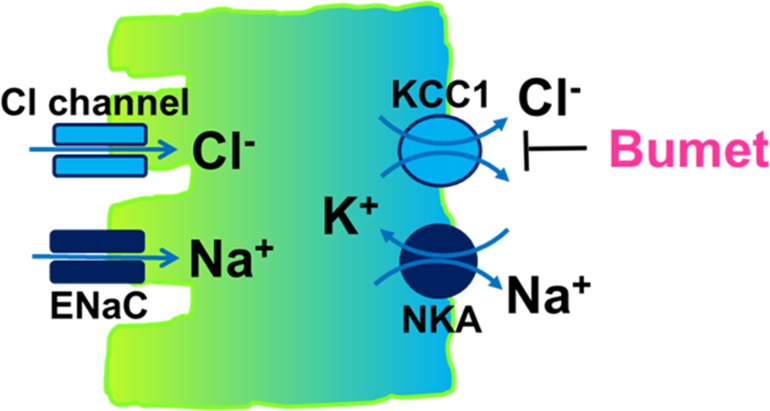
Transport model for electrogenic Cl^-^ absorption in surface cell of late distal colonic epithelium. Cl^-^ enters via a Cl^-^ channel conductance and egresses through a bumetanide-sensitive K^+^-Cl^-^ cotransporter. K^+^ is recycled uphill into the cell by Na^+^,K^+^-ATPase (NKA), while the latter pumps Na^+^ that enters via ENaC against electrochemical gradient out of the cell. Thus, transepithelial Na^+^ and Cl^-^ absorption are both an active process and operate in parallel.

In these present studies, we presented evidence that an alternative Cl^-^ absorptive mechanism exists in the late distal colon. This Cl^-^ absorptive mechanism is active and electrogenic; it operates in parallel with ENaC-mediated Na^+^ absorption, but does not depend on ENaC activity, as proposed per current model [2]. Also in support of electrogenic Cl^-^ absorption in this colonic segment is the observation that the late distal colon is tighter than its early counterpart. This is evidenced by higher electrical resistance and lower lumen negative potential difference and I_sc_ (see Table 1). Given its sensitivity to apical NPPB/glibenclamide and basolateral bumetanide, we proposed a transport model for this alternative Cl^-^ absorption process (see Fig 9).

According to this new model, Na^+^ and Cl^-^ move in parallel and are both actively absorbed. The energy that drives Na^+^ and Cl^-^ uptakes is derived from the basolateral Na^+^,K^+^-ATPase (NKA). Through active pumping of Na^+^ out of the cell and K^+^ into the cell, NKA lowers intracellular Na^+^ concentration ([Na^+^]_i_) and elevates intracellular K^+^ concentration ([K^+^]_i_), generating two chemical gradients across cell membrane: the gradient for Na^+^ and the gradient for K^+^. Whereas the gradient for Na^+^ enables apical ENaC to move Na^+^ from the lumen into the cell, the gradient for K^+^ provides the driving force for active uptake of Cl^-^ via the secondary active basolateral bumetanide-sensitive KCl cotransporter [24] (likely KCC1 [25]), which extrudes one K^+^ with one Cl^-^ from the cell to the blood, and so lowers [Cl^-^]_i_; the apical NPPB/glibenclamide-sensitive Cl^-^ conductance (likely a Cl^-^ channel/transporter) subsequently moves Cl^-^ from the lumen into the cell.

It is worth noting, however, that the model described in Fig 9 is the simplest one. It describes the basic components required for active uptake of Cl^-^ in this part of the colon. Other transporters may be involved to facilitate this transport process. These may include: 1) basolateral K^+^ channel. This K^+^ channel may participate in the fine-tuning of the activity of KCC1 through adjusting [K^+^]_i_; 2) basolateral Cl^-^/ HCO_3_^-^ exchange. There are reports that this anion exchange may contribute to Cl^-^ secretion [13] and Cl^-^ absorption [2]; 3) apical Na^+^-Cl^-^ cotransporter (NCC) [26]. NCC transcript was expressed in late distal colonic mucosa (see Table 3 and Fig 6). NCC is likely present at the apical membrane of distal colonic epithelial cells, facilitating Cl^-^ entry, particularly when [Cl^-^]_L_ is low; 4) apical DRA [27]. As a member of the SLC26 family, this Cl^-^/HCO_3_^-^ exchanger is reported to have concurrent conductive Cl^-^ activity [28]. Thus, it may help provide an additional pathway or source of energy to take up Cl^-^ from the lumen when [Cl^-^]_L_ is low through using the gradient of HCO_3_^-^ generated internally through the hydration of CO_2_ by carbonic anhydrase [29–31].

In sweat duct, frog skin, and other high-resistance (400 ohm.cm^2^ or higher) epithelia, Cl^-^ absorption is achieved primarily through coupling to active Na^+^ absorption [10, 32]. In these non-leaking epithelia, a separate active mechanism for Cl^-^ absorption is biophysically unnecessary. Compared to these tight epithelia, the resistance of colon epithelium is much lower (150 ohm.cm^2^ or lower) (see Table 1; also refs [11, 15]). While being leaky may confer to this colonic epithelium the ability to perform other beneficial functions such as absorbing luminal nutrients and monitoring luminal microbiota, it also makes it difficult to simply couple Cl^-^ absorption passively to Na^+^ absorption. To achieve the goal of regulating salt homeostasis in this physiologically important epithelium, a parallel active Cl^-^ absorption is necessary. This active Cl^-^ absorption differs from the transcellular Cl^-^ absorption that couples to ENaC in the sweat duct. It also differs from the Cl^-^ absorption that occurs via a paracellular Cl^-^ shunt in leaky proximal tubule and other intestinal epithelia. This active Cl^-^ flux runs in parallel with ENaC (see Fig 9). Thus, the presence of this active Cl^-^ transport would best fulfill the function of the colon by this epithelium.

Like ENaC, this active Cl^-^ absorption is regulated in accordance with the salt status of the body. Consequently, when NaCl is in excess, e.g., when dietary salt intake is increased, it is down regulated (see Fig 8). In contrast, when NaCl is in deficit (i.e., when dietary salt intake is decreased or in pathological situations such as in diarrhea or diuresis in which high salt loss occurs), it is up regulated (see Fig 8). In response to salt depletion, aldosterone is secreted [33], which then stimulates electrogenic Na^+^ absorption in the distal colon as well as the distal nephron via up regulation of ENaC, NKA, and their regulators such as SGK and CHIF [33]. Whether aldosterone stimulates parallel electrogenic Cl^-^ absorption is unknown; also uncertain is whether this occurs via the up-regulation of CFTR in the ENaC-expressing absorbing surface epithelium of the distal colon/nephron. In addition to aldosterone, the extracellular calcium-sensing receptor (CaSR) is another key receptor mechanism that regulates homeostasis of salt transport, particularly in the gut, where CaSR coordinates monovalent and divalent ion homeostasis [34–36]. Increases of luminal as well as serosal [Ca^2+^] and CaSR activity decrease electrogenic Cl^-^ secretion [15, 18, 37, 38]; they also increase electroneutral NaCl absorption [11, 38]. The goal is to ensure, along with CaSR-mediated regulation in parathyroid and kidney, that extracellular [Ca^2+^] is stable without significant elevations. Whether CaSR increases electrogenic Na^+^ and Cl^-^ absorption in this highly regulated late distal colon remains to be determined.

Previous studies have shown significant segmental heterogeneity between the proximal and distal colon in terms of ion transport function and sensitivity to aldosterone stimulation. For example, basal K^+^ movement is secretory (via apical K^+^ channel conductance [39]) in the proximal colon but is absorptive (via apical H^+^,K^+^-ATPase [40]) in the distal colon. Their responses to aldosterone stimulation and dietary K^+^ loading also differ, with enhancing K^+^ secretion in the proximal colon versus converting K^+^ movement from absorption to secretion in the distal colon [41]. Similar differences are also seen in Na^+^ transport. Without aldosterone the basal Na^+^ absorption is electroneutral (via NHE3) in the proximal colon and is electroneutral (via NHE3) and electrogenic (via ENaC) in the distal colon [2, 42]. Aldosterone stimulation enhances NHE3-mediated Na^+^ transport in the proximal colon [43], but causes a flip of Na^+^ transport from NHE3 to ENaC in the distal colon [44]. Consistent with this aldosterone flip, our functional studies in the distal colon revealed a significant increase in ENaC current (2-fold increase in early distal colon and 5-fold increase in late distal colon, P<0.01) in response to dietary Na^+^ depletion and a significant decrease (10-fold decrease in the early distal colon and 7-fold decrease in the late distal colon, P<0.01) in response to dietary Na^+^ repletion (Table 4). It would be interesting to know if these dietary treatments cause inverse changes in NHE3 function.

In the present study, we show that a discrepancy also exists within the distal colon between the early and the late distal colon segments. We found that while the early distal colon was predominantly salt secretory, the late distal colon was primarily salt absorptive. Accordingly, inversed profiles of ion transporters for secretion and for absorption were seen, with CFTR, NKCC1 and SBC1 being more abundant in the early distal colon than the late distal colon, and ENaC and KCC1 being prominent in the late distal colon and negligible in the early distal colon (see Table 3, Fig 6 and Fig 7). Consistent with this arrangement, we found that, although both currents are present in both segments, the Cl^-^ secretory I_sc_, which, in current direction, is inhibitory by bumetanide, was primarily localized to the salt secretory early distal colon, whereas the Cl^-^-absorptive I_sc_, which, in current direction, is stimulatory by bumetanide, was primarily present in the salt absorptive late distal colon (see Fig 1). The results from our physiological study have confirmed this functional arrangement. Accordingly, secretagogues (e.g., forskolin) stimulate salt secretion primarily in the early distal colon (see Fig 2), whereas aldosterone/low salt diet intake stimulates salt absorption mainly in the late distal colon (see Fig 8). Thus, the cycling of fluid and electrolytes through segregated secretion and absorption appears to be a common theme in the mammalian intestine.

In summary, our present studies demonstrate the following: 1) rat distal colonic epithelium is able to both actively secrete and absorb Cl^-^, with secretion primarily occurring in ENaC-absent early distal colon and absorption predominantly in ENaC-expressing late distal colon; 2) active Cl^-^ absorption in the late distal colon is increased by dietary Cl^-^ (salt) depletion and decreased by dietary Cl^-^ (salt) repletion; and 3) active Cl^-^ absorption occurs through transcellular transport that involves an apical NPPB/glibenclamide-sensitive Cl^-^ conductance and a basolateral bumetanide-sensitive K^+^-Cl^-^ cotransporter. We conclude that an electrogenic Cl^-^ extrusion mechanism exists and operates in parallel with the electrogenic ENaC in the distal colon (and probably in nephron as well) where the final control of NaCl absorption occurs before effluents are discharged from the body.
